# Mechanistic Insights into Glycerol Oxidation to High-Value Chemicals via Metal-Based Catalysts

**DOI:** 10.3390/molecules30061310

**Published:** 2025-03-14

**Authors:** Junqing Li, Ying Tu, Kelin He, Chao Chen, Lixing Liang, Chongze Ruan, Qitao Zhang

**Affiliations:** 1 International Collaborative Laboratory of 2D Materials for Optoelectronics Science and Technology of Ministry of Education, Institute of Microscale Optoelectronics, Shenzhen University, Shenzhen 518000, China; lijunqing2022@email.szu.edu.cn (J.L.); arckytu@outlook.com (Y.T.); chenchao@szu.edu.cn (C.C.); 2 College of Materials and Energy, South China Agricultural University, Guangzhou 510642, China; 13450137320@163.com (L.L.); rcz13266465725@163.com (C.R.)

**Keywords:** metal-based catalysts, glycerol selectivity oxidation, high-value chemicals, mechanistic pathways

## Abstract

The oxidation of glycerol offers a valuable route for producing high-value chemicals. This review provides an in-depth analysis of the current advancements and mechanistic insights into novel metal-based catalysts for glycerol oxidation. We discuss the catalytic roles of both precious metals (e.g., Pt, Pd, Au), noted for their high efficiency and selectivity, and cost-effective alternatives, such as Ni, Cu, and Fe. Bimetallic and metal oxide catalysts are highlighted, emphasizing synergistic effects that enhance catalytic performance. This review elucidates the key mechanism involving selective adsorption and oxidation, providing detailed insights from advanced spectroscopic and computational studies into the activation of glycerol and stabilization of key intermediates, including glyceraldehyde and dihydroxyacetone. Additionally, selective carbon–carbon bond cleavage to yield smaller, valuable molecules is addressed. Finally, we outline future research directions, emphasizing the development of innovative catalysts, deeper mechanistic understanding, and sustainable process scale-up, ultimately advancing efficient, selective, and environmentally friendly catalytic systems for glycerol valorization.

## 1. Introduction

Glycerol, also known as glycerin, is a trihydroxy alcohol with the chemical formula C_3_H_8_O_3_. It is predominantly produced as a byproduct in the biodiesel industry during the transesterification process of fats and oils [1,2,3,4,5]. This process generates approximately 10% glycerol as a byproduct. The substantial production of biodiesel has led to an excess supply of glycerol, creating both economic challenges and opportunities. Economically, the oversupply of glycerol has driven down market prices, making it an attractive, low-cost feedstock for various chemical transformations [6,7,8,9,10]. This situation aligns well with sustainable development principles, offering a renewable alternative to traditional fossil fuel-based feedstocks [11,12,13,14]. Utilizing glycerol to produce high-value chemicals not only adds economic value but also addresses waste disposal issues, contributing to a more circular economy [15,16,17]. From an environmental perspective, converting glycerol into value-added products is a prime example of green chemistry in action [18,19,20,21,22]. This process reduces waste and minimizes the environmental impact associated with biodiesel production. The versatile chemical structure of glycerol, featuring three hydroxyl groups, makes it an excellent candidate for a variety of chemical reactions, including oxidation, reduction, etherification, and esterification [23,24,25,26]. By leveraging glycerol’s potential as a building block for these reactions, the chemical industry can advance towards more sustainable and eco-friendly practices [27,28,29,30].

The oxidation of glycerol can yield several high-value chemicals, each with significant industrial applications. Dihydroxyacetone (DHA), for instance, is widely used in the cosmetic industry, particularly in sunless tanning products, and serves as a precursor for synthesizing other fine chemicals [31,32,33,34]. Glyceraldehyde (GA) finds applications in biochemical research and as an intermediate in pharmaceutical and specialty chemical synthesis [35,36,37,38]. Formic acid (FA) is employed as a preservative and antibacterial agent in livestock feed and various synthesis processes [25,39,40,41,42]. Glyceric acid (GLA) is used in pharmaceuticals, cosmetics, and as a precursor for biodegradable polymers. Additionally, oxalate is utilized in the manufacture of cleaning agents, metal polishing, and as a stabilizing agent in the pharmaceutical industry [43,44,45,46]. These examples highlight the commercial potential of glycerol oxidation, underscoring the importance of developing efficient catalytic processes [47,48,49].

**Figure 1:**
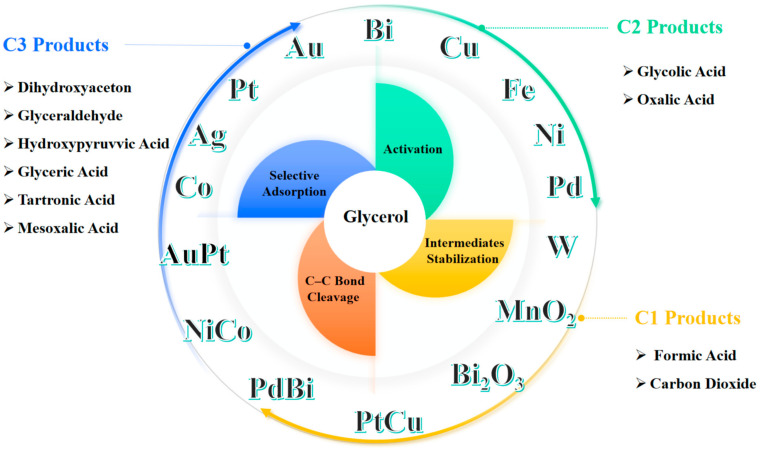
Overview of metal catalysts in glycerol oxidation and its high-value products.

The primary objective of this review is to explore and summarize the current advancements and mechanistic insights into metal-based catalysis for glycerol oxidation. As summarized in Figure 1, this review will examine various types of metal catalysts, including monometallic catalysts, bimetallic catalysts and metal oxide catalysts. Understanding the mechanistic roles of these catalysts in glycerol oxidation is crucial, as it involves selective adsorption, activation of glycerol, stabilization of intermediates, and selective C–C bond cleavage. The efficiency and selectivity of different catalysts in producing specific C1–C3 oxidation products will also be highlighted. By providing a comprehensive overview of the current state of research in metal-catalyzed glycerol oxidation, this review aims to identify key challenges and suggest future research directions. Enhancing the efficiency and selectivity of these catalytic processes is vital for both economic and environmental sustainability, offering significant potential for industrial applications and the development of greener chemical processes.

## 2. Glycerol Oxidation: Basics and Catalysts

The oxidation of glycerol presents a valuable pathway for the synthesis of numerous high-value chemicals. This process involves the transformation of glycerol’s hydroxyl groups through various mechanistic pathways, facilitated by metal-based catalysts [50,51,52,53]. By understanding and optimizing these oxidation pathways, it is possible to selectively produce a range of industrially important products, such as glyceraldehyde, dihydroxyacetone, and glyceric acid [54,55,56]. The selective oxidation of glycerol not only adds value to this abundant byproduct of biodiesel production but also contributes to the advancement of sustainable chemical processes.

### 2.1. Glycerol Properties and Oxidation Products

#### 2.1.1. Chemical Structure and Reactivity

Glycerol consists of a three-carbon chain with each carbon atom bonded to a hydroxyl group (-OH), making it a trihydroxy alcohol. The chemical structure of glycerol can be represented as HOCH_2_-CHOH-CH_2_OH [6,57,58,59]. This molecular structure imparts glycerol with its characteristic properties of being highly hygroscopic, viscous, and miscible with water and alcohols. The reactivity of glycerol is primarily due to the presence of these three hydroxyl groups [60,61,62,63]. These -OH groups make glycerol an excellent candidate for various chemical reactions, including oxidation, reduction, esterification, and etherification. In oxidation reactions, the hydroxyl groups can be selectively oxidized to form a range of valuable products [64,65,66]. The primary alcohol groups (on C1 and C3) and the secondary alcohol group (on C2) present distinct reactive sites that can lead to different oxidation pathways and products [41,67,68].

#### 2.1.2. Effect of Acid and Base in Glycerol Oxidation

The oxidation of glycerol is significantly influenced by the reaction conditions, especially the presence of acids or bases. Bases, in particular, play a critical role in promoting glycerol oxidation through several mechanistic pathways. In basic conditions, alcohol groups on glycerol molecules are readily deprotonated to form electron-rich alkoxide species, which are more susceptible to oxidation [69]. This process increases both the rate and efficiency of glycerol oxidation reactions, facilitating the selective activation and transformation of glycerol into specific oxidation products. Importantly, the presence of a base also mediates the reversible interconversion between two critical oxidation intermediates: dihydroxyacetone (DHA) and glyceraldehyde (GAL). Under basic conditions, DHA and GAL rapidly interconvert through an enediol intermediate (HO–CH=CH–OH) [70]. This base-catalyzed equilibrium significantly affects the distribution and selectivity of final oxidation products, as different reaction conditions and catalyst compositions may shift the equilibrium toward either DHA or GAL, thereby determining subsequent oxidation pathways. Conversely, acidic conditions typically favor specific reaction pathways and product selectivity by stabilizing certain intermediates and facilitating dehydration and rearrangement reactions. Acidic media may suppress the formation of alkoxides, instead promoting the protonation of reactive intermediates, thereby altering oxidation mechanisms and product distributions compared to those under basic conditions [71]. Overall, understanding and controlling acid–base conditions are essential for optimizing catalyst performance, reaction selectivity, and product yields in glycerol oxidation processes.

#### 2.1.3. Catalytic Mechanisms in Glycerol Oxidation: Thermal, Photocatalytic, and Electrocatalytic Approaches

Glycerol oxidation mechanisms vary significantly depending on the energy source employed: thermal catalysis, photocatalysis, or electrocatalysis. In thermal catalysis, heat is the primary driver, where elevated temperatures (typically 60–120 °C or higher) significantly enhance the reaction rates [72]. Thermal energy promotes the adsorption of glycerol and oxidizing species onto catalyst surfaces, facilitating electron transfer and oxidative transformations. Catalysts, typically composed of metal nanoparticles supported on stable substrates, activate glycerol through adsorption, formation of reactive intermediates, and subsequent electron transfer steps, resulting in the cleavage of C–H and O–H bonds and, ultimately, carbon–carbon bond cleavage under suitable conditions [73].

Photocatalysis, in contrast, utilizes photons, typically from UV or visible light, to activate semiconductor catalysts. When the catalyst absorbs photons with energy equal to or higher than its bandgap, electron–hole pairs are generated. These photo-induced holes (h^+^) act as powerful oxidizing agents, directly oxidizing adsorbed glycerol molecules or water to generate hydroxyl radicals (•OH) [74]. These highly reactive radicals or the holes themselves initiate the oxidative processes by abstracting electrons or protons from glycerol, facilitating conversion into oxidation intermediates or complete oxidation products at relatively mild ambient conditions, often at room temperature.

Electrocatalysis leverages electrical energy applied to electrodes within electrochemical cells, operating under mild and precisely controlled conditions. Oxidation occurs specifically at the anode, where glycerol molecules lose electrons under externally applied potentials, generating oxidation products through multiple intermediate states [75]. The electrode potential significantly influences reaction selectivity and efficiency, providing unique opportunities to selectively produce desired oxidation intermediates by carefully tuning the potential applied. Typical conditions involve aqueous electrolytes and mild reaction temperatures, usually near ambient conditions.

Each catalytic method, thus, offers distinct mechanistic features and operational advantages: thermal catalysis offers reliability, maturity, and industrial scalability with high reaction rates [76]; photocatalysis provides an environmentally sustainable and energy-efficient alternative under ambient conditions; electrocatalysis offers high product selectivity, controllability, and excellent compatibility with renewable electricity sources [77]. Understanding these unique mechanistic aspects and conditions allows for optimized and informed choices in catalytic strategy selection for targeted glycerol oxidation applications.

#### 2.1.4. Oxidation Pathways and Products

**Figure 2:**
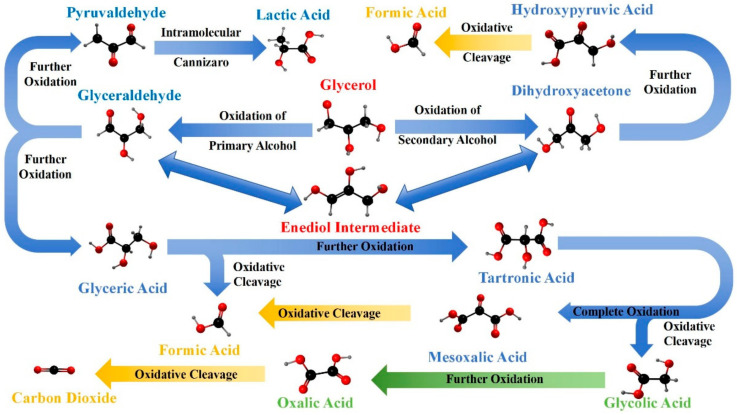
Oxidation pathways of glycerol and resulting C1 (yellow), C2 (green), and C3 (blue) products.

The oxidation of glycerol can proceed through multiple pathways, leading to a variety of C1–C3 oxidation products, as illustrated in Figure 2, each with significant industrial applications [67,78,79,80]. The selectivity of these pathways can be influenced by the choice of catalyst, reaction conditions, and the presence of specific promoters or inhibitors [81,82].

Initially, one of the primary hydroxyl groups (-CH_2_OH) of glycerol can be selectively oxidized to form glyceraldehyde, a molecule that contains both aldehyde and hydroxyl functional groups, serving as a crucial intermediate in carbohydrate metabolism [2,83,84,85]. Alternatively, the oxidation can target the secondary hydroxyl group (-CHOH-), converting it into a ketone group (=C=O) and producing dihydroxyacetone, the simplest ketose sugar, with significant applications in cosmetics, particularly in sunless tanning products [44,86,87,88].

As the oxidation process continues, glyceraldehyde can be further oxidized to glyceric acid, where the aldehyde group is transformed into a carboxylic acid group (-COOH), resulting in an α-hydroxy acid involved in various metabolic pathways. Dihydroxyacetone, on the other hand, can be oxidized to hydroxypyruvic acid, introducing a carboxylic acid group adjacent to the ketone, thus forming an intermediate, significant in amino acid metabolism [40,89,90,91]. Progressing further, glyceric acid may undergo additional oxidation to form tartronic acid, where an additional carboxyl group is added to the molecule, yielding an α-hydroxy-dicarboxylic acid known for its chelating properties [3,50,92]. The subsequent oxidation of tartronic acid leads to mesoxalic acid, where the hydroxyl group is fully oxidized to a carbonyl group, producing an unstable tricarboxylic acid [16,18,19,93].

Significantly, glyceraldehyde also serves as a key intermediate to produce lactic acid. Under suitable catalytic conditions, glyceraldehyde undergoes dehydration to form pyruvaldehyde (methylglyoxal). This intermediate subsequently participates in a benzilic acid rearrangement, where intramolecular rearrangement occurs, resulting in the formation of lactic acid, an important chemical extensively used in the food, pharmaceutical, and polymer industries [76]. This rearrangement involves the migration of an alkyl group accompanied by the rearrangement of electrons, resulting in a conversion from the ketone (pyruvaldehyde) to the carboxylic acid functionality of lactic acid. It is worth noting that glycerol can also indirectly be oxidized to acrylic acid, a valuable chemical widely used in polymer industries. This indirect pathway typically involves the acid-catalyzed dehydration of glycerol to acrolein, followed by oxidation to acrylic acid [94].

Under harsher oxidative conditions, especially with strong oxidizing agents like potassium permanganate or nitric acid, the glycerol molecule can experience oxidative cleavage of its carbon–carbon bonds [7,8,15,43,95]. This leads to the formation of smaller molecules such as oxalic acid, a strong dicarboxylic acid commonly found in plants, and formic acid, the simplest carboxylic acid noted for its antimicrobial properties [6,43,96]. These cleavage reactions highlight the susceptibility of the glycerol backbone to overoxidation, especially when reaction conditions are not carefully controlled [24,97,98,99]. Ultimately, the complete oxidation of glycerol results in the formation of carbon dioxide and water, representing the end products of organic carbon oxidation and contributing to the global carbon cycle [19,100,101,102,103,104]. These transformations highlight the potential of glycerol to form both simple and complex molecules through oxidation processes.

These oxidation products exemplify the potential of glycerol as a versatile feedstock for producing commercially valuable chemicals. Developing efficient and selective catalytic processes for glycerol oxidation is crucial to maximize the yield of desired products and minimize the formation of undesired byproducts [105,106,107,108]. The following sections will delve into the various types of metal-based catalysts used in glycerol oxidation and their roles in facilitating these transformations.

In addition, this review also provides more detailed information for each composition regarding the analysis techniques used to monitor the oxidation byproducts (such as chromatography, infrared, spectrophotometry, etc.). However, it is crucial to acknowledge potential analytical challenges, particularly in the quantification and identification of glycerol oxidation products using HPLC. One significant issue is the accidental overlap of peaks, which can lead to misidentification. For instance, formic acid and dihydroxyacetone have been reported to share identical retention times on certain ion-exclusion columns, resulting in inaccurate analytical results [109]. To mitigate such errors, alternative analytical methods or complementary columns should be employed to ensure accurate product identification.

Following this discussion, Table 1 summarizes the analytical techniques, exact percentages of oxidation byproducts, glycerol oxidation efficiencies, and dominant oxidation products across different catalyst types. The extent of glycerol oxidation demonstrated is significant for practical applications, indicating strong industrial applicability and efficiency. Furthermore, the dominant oxidation product significantly surpasses other byproducts, clearly demonstrating the selectivity of these catalytic systems toward valuable chemicals like DHA and FA.

### 2.2. Types of Metal-Based Catalysts

Metal-based catalysts are crucial in the oxidation of glycerol, offering a range of properties that enhance activity, selectivity, and stability [105,110,112]. These catalysts can be broadly categorized into monometallic catalysts, bimetallic catalysts, and metal oxide catalysts [111,113,114]. Each category has distinct advantages and specific applications in glycerol oxidation, contributing to the formation of various high-value chemicals [115,116].

#### 2.2.1. Monometallic Catalyst

**Figure 3:**
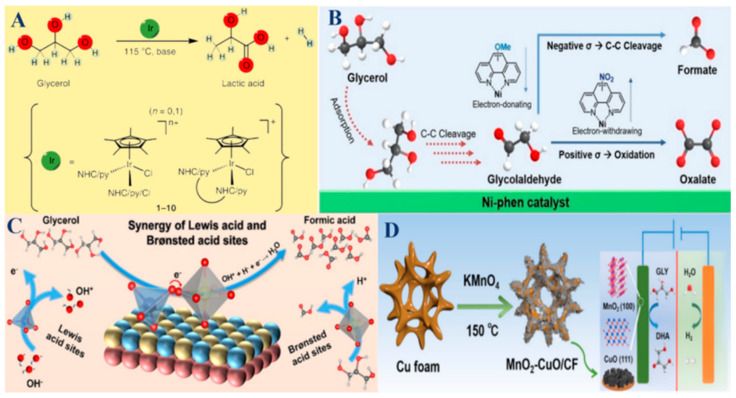
(**A**) Iridium-catalyzed conversion of glycerol to LA. Reproduced with permission: Copyright 2014, Springer Nature [44]. (**B**) Schematic illustration of the glycerol oxidation pathway and its correlation with the Hammett parameters. Reproduced with permission: Copyright 2023, Wiley-VCH [57]. (**C**) The proposed synergistic mechanism of GOR to FA catalyzed by NiCo_2_O_4_/NF with regulable Lewis and Brønsted acid sites. Reproduced with permission: Copyright 2024, Wiley-VCH [65]. (**D**) Schematic of the synthesis of MnO_2_-CuO/CF and the application in GOR and HER. Reproduced with permission: Copyright 2024, Elsevier [92].

Monometallic catalysts consist of a single type of metal, such as noble metals (Pt, Pd, Ir, or Au) and non-noble metals (Bi, Co, Ni, Cu, or Fe) [2,8,39,43,44,57,83,95,99]. As shown in the schematic illustration in Figure 3A,B, Ir- and Ni-based catalysts both effectively drive the selective oxidation of glycerol [44,57]. These metal catalysts are highly active and selective due to the intrinsic properties of the metals, while non-precious metals offer a cost-effective alternative to precious metals, with considerable catalytic activity and selectivity for glycerol oxidation [41,100,107,117]. However, monometallic catalysts can suffer from limitations such as deactivation due to poisoning by reaction intermediates and a lack of flexibility in tuning catalytic properties to optimize both activity and selectivity. Despite these challenges, their high catalytic efficiency and well-understood mechanisms make them valuable in fundamental research and specific industrial applications.

#### 2.2.2. Bimetallic Catalysts

Bimetallic catalysts combine two different metals, exploiting the synergistic effects that enhance catalytic performance beyond what is possible with monometallic catalysts. These combinations can significantly improve activity, selectivity, and stability. For instance, some Pt-based bimetallic catalysts, like Pt-Bi, Pt-Cu, Pt-Au, and Pt-Sb, show enhanced glycerol adsorption and activation, leading to higher yields of DHA and GLA compared to their monometallic counterparts [98,111,114,115]. The interaction between the two metals can also reduce catalyst deactivation, improve resistance to poisoning, and allow for the fine-tuning of electronic and geometric properties. Similarly, some Pd-based bimetallic catalysts, such as Pd-Bi, Pd-Sn, and Pd-Pt, benefit from enhanced electronic interactions, resulting in better performance for selective oxidation processes [67,78,104]. Non-noble metal bimetallic systems, such as Ni-Mo and Ni-Co, offer cost-effective solutions with significant improvements in catalytic efficiency and selectivity [61,64,65]. As illustrated in Figure 3C, the proposed synergistic mechanism of GOR to FA is catalyzed by NiCo_2_O_4_/NF with regulable Lewis and Brønsted acid sites [65].

#### 2.2.3. Metal Oxide Catalysts

For instance, the synthesis of the MnO_2_-CuO/CF catalyst and its application in GOR and HER were achieved (Figure 3D) [92]. Many metal oxide catalysts, including transition metal oxides, like BiVO_4_, TiO_2_, Fe_2_O_3_, MnO_2_, WO_3_, CuO, Co_3_O_4_, VOSO_4_ and H_5_PV_2_Mo_10_O_40_, offer unique properties that make them effective in glycerol oxidation [3,24,39,40,51,59,92,110,118,119]. These catalysts are known for their high surface area, stability, and the ability to generate oxygen vacancies, which facilitate oxidation reactions. For example, BiVO_4_ photoanodes enhance light absorption and photocurrent density, making them highly effective in photocatalytic glycerol oxidation to produce DHA [59,79]. The ability to generate in situ reactive oxygen species, such as H_2_O_2_ in the Fenton process, further enhances their catalytic performance. While metal oxide catalysts may not always match the activity levels of monometallic or bimetallic catalysts, their robustness, cost-effectiveness, and environmental compatibility make them attractive for sustainable industrial processes.

## 3. Mechanistic Roles of Metal-Based Catalysts in Glycerol Oxidation

Metal-based catalysts play essential roles in the oxidation of glycerol, facilitating its transformation into valuable chemicals through various mechanistic pathways. These catalysts enhance the activation of glycerol by promoting the dehydrogenation of hydroxyl groups, forming key intermediates such as glyceraldehyde and dihydroxyacetone [110,116,120]. Additionally, they stabilize these intermediates, preventing overoxidation and ensuring efficient conversion into desired products. Selective adsorption and oxidation mechanisms, particularly involving catalysts like silver nanoparticles and bismuth-modified surfaces, improve the selective interaction with specific hydroxyl groups, enhancing reaction efficiency and selectivity. Moreover, metal catalysts such as nanoporous BiVO_4_ and single Ni atoms facilitate selective carbon–carbon (C–C) bond cleavage, generating smaller, high-value molecules like glycolaldehyde. Understanding these mechanistic roles is crucial for optimizing catalytic systems, advancing sustainable chemical processes and enhancing the economic and environmental benefits of glycerol utilization.

### 3.1. Selective Adsorption and Oxidation

Selective adsorption and oxidation play a pivotal role in the catalytic conversion of glycerol into valuable chemical products. This process involves the precise interaction between glycerol molecules and the catalytic surface, facilitating targeted oxidative transformations [11,80,82]. By enhancing the adsorption of specific hydroxyl groups within the glycerol molecule, catalysts can significantly improve the efficiency and selectivity of the oxidation process. This section delves into the mechanisms by which metal catalysts, particularly those involving silver nanoparticles and bismuth-modified surfaces, promote selective adsorption and oxidation. Understanding these mechanisms is crucial for optimizing catalyst design and reaction conditions to achieve high selectivity and yields of desired products such as DHA and other industrially relevant chemicals [85,88,91]. Through a detailed examination of recent advancements and experimental findings, we aim to provide insights into the intricate dynamics of selective adsorption and oxidation, underscoring their importance in the broader context of glycerol valorization and sustainable chemical production.

**Figure 4:**
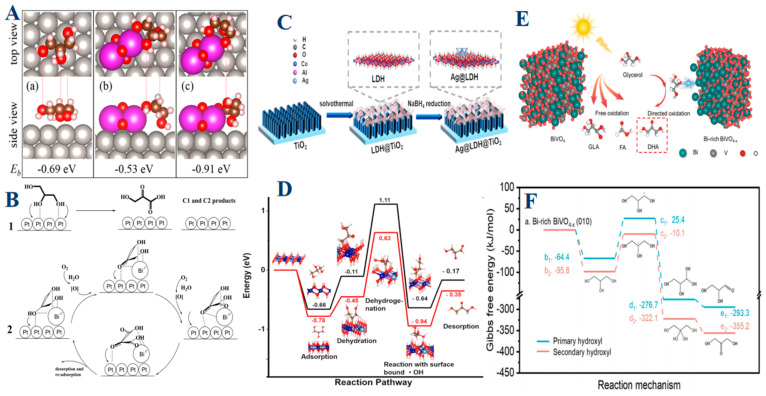
(**A**) Top and side views of glycerol adsorption structures on Pt (111) (a), Pt (111)-Bi_2_O_3_ (b and c), along with the binding energy (E_e_). (**B**) Proposed mechanism of glycerol oxidation over (1) 3Pt/SBA-15 and (2) 3Pt-0.3Bi/SBA-15. Reproduced with permission: Copyright 2020, American Chemical Society [111]. (**C**) The Ag@LDH@TiO_2_ photoanode is made with Ag nanoparticles in LDH nanosheets grown on TiO_2_ arrays, (**D**) Free energy profile of glycerol oxidation on the Co_6_Al_2_(OH)_16_ model. Reproduced with permission: Copyright 2022, American Chemical Society [1]. (**E**) Schematic illustration of PEC glycerol oxidation to DHA using a Bi-rich BiVO_4-x_ photoanode, (**F**) The Gibbs free energy profiles for oxidation processes involving primary and secondary hydroxyl groups on Bi-rich BiVO_4-x_ surfaces. Reproduced with permission: Copyright 2024, Springer Nature [16].

In the Pt-Bi catalyst system, the introduction of Bi significantly alters the surface properties of platinum (Pt), resulting in a selective adsorption mechanism that favors the secondary hydroxyl group of glycerol [111]. This selective adsorption is crucial for guiding the oxidation process toward DHA, the most desirable product due to its high market value and robust carbon chain. Moreover, the catalytic activity is enhanced as the Pt-Bi combination promotes the oxidation of the secondary hydroxyl group more effectively than the primary groups. To further improve selectivity, Bi plays a protective role by blocking higher-energy Pt sites that are prone to overoxidation and C–C bond cleavage (Figure 4A,B). This blocking effect not only enhances selectivity but also prevents the formation of unwanted byproducts, ensuring a high yield of DHA. Building on the concept of selective adsorption, the Ag@LDH@TiO_2_ photoanode represents a more complex catalytic system, where silver nanoparticles (Ag) are supported on layered double hydroxide (LDH) nanosheets, anchored to a TiO_2_ substrate [1]. In this system, LDHs are critical for the selective adsorption of the secondary hydroxyl group of glycerol, a key step in DHA formation. Complementing this selective adsorption, the silver nanoparticles enhance the kinetics of the oxidation process, accelerating the conversion of glycerol to DHA. Additionally, the system efficiently mediates the generation and activity of hydroxyl radicals, crucial oxidizing agents. By controlling these radicals’ activity, the Ag@LDH@TiO_2_ system ensures that the oxidation process remains selective and controlled, avoiding overoxidation and thereby increasing the yield and purity of DHA (Figure 4C,D). Further expanding on the importance of surface engineering, the BiVO_4_ photoanode system demonstrates how surface modifications can significantly impact catalytic performance (Figure 4E). By introducing bismuth-rich domains and oxygen vacancies, the BiVO_4_ surface is engineered to enhance the selective adsorption of glycerol’s secondary hydroxyl groups, driving the oxidation process towards DHA production with remarkable efficiency [16]. This engineered surface not only boosts photocurrent density, crucial for the photoelectrochemical (PEC) process, but also dramatically increases DHA selectivity. These enhancements are achieved by suppressing unwanted side reactions, particularly overoxidation, which can lead to carbon chain breakdown and the formation of less valuable products. Additionally, the Bi-rich surface improves charge separation and elevates surface potential, further enhancing the overall efficiency and yield of DHA.

In summary, the meticulous engineering of metal-based surfaces is fundamental to optimizing the selective oxidation of glycerol. By carefully tailoring surface properties, researchers can significantly influence the reaction pathway, steering it toward the production of dihydroxyacetone (DHA), the most valuable product in this process. Whether through the strategic addition of bismuth in platinum catalysts, the integration of silver nanoparticles within layered double hydroxide (LDH) systems, or the precise surface engineering of BiVO_4_ photoanodes, each approach exemplifies how targeted modifications can enhance both reaction kinetics and selectivity. These advancements not only drive the desired chemical transformations more efficiently but also suppress unwanted side reactions that could otherwise reduce product yield and compromise stability (Figure 4F). As a result, metal-based catalysts emerge as indispensable tools in the efficient and selective conversion of glycerol into high-value chemicals, offering significant potential for both industrial applications and broader catalytic innovations. This comprehensive understanding underscores the critical role of surface engineering in advancing the field of glycerol oxidation and highlights the promising future of metal-based catalysis in sustainable chemical production.

### 3.2. Activation of Glycerol

The activation of glycerol is a crucial step in the oxidation process, serving as the foundation for subsequent transformations into valuable chemicals. Glycerol requires activation to facilitate its conversion into more complex products. This activation typically involves the initial dehydrogenation of glycerol’s hydroxyl groups, which is essential for creating reactive intermediates such as glyceraldehyde and dihydroxyacetone. These intermediates play pivotal roles in the formation of higher-value chemicals like glyceric acid and hydroxypyruvic acid. The efficiency of this activation step greatly influences the overall selectivity and yield of the desired oxidation products. Metal catalysts, such as platinum and defective manganese dioxide, have been shown to enhance this activation process by facilitating the necessary C–H and O-H bond activations. By improving the activation of glycerol, these catalysts enable more efficient and selective oxidation pathways, thereby increasing the economic viability and sustainability of converting glycerol into industrially important chemicals.

**Figure 5:**
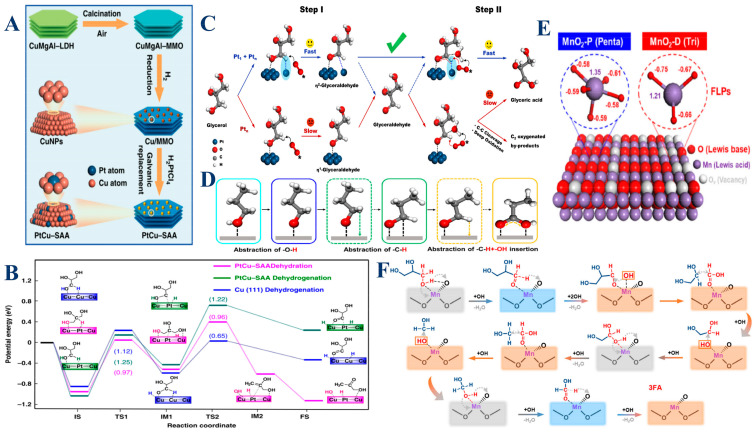
(**A**) A schematic illustration of the preparation of PtCu–SAA. Catalytic evaluation of PtCu-SAA and monometallic catalysts (Pt/MMO and Cu/MMO) for glycerol hydrogenolysis to 1,2-PDO. (**B**) Potential energy profiles for glycerol hydrogenolysis on Cu (111) and PtCu–SAA (111) surfaces. Reproduced with permission: Copyright 2019, Springer Nature [115]. (**C**) The proposed cascade synergistic catalysis strategy utilizes atomic Pt_1_ and cluster Pt_n_ sites for the selective oxidation of glycerol to GLYA as a showcase. (**D**) The proposed surface reaction process for the oxidation of 1-propanol to propionic acid. Reproduced with permission: Copyright 2022, Springer Nature [107]. (**E**) Structural diagram and Mulliken charge distribution of the Frustrated Lewis Pair in MnO_2_-P and MnO_2_-D, (**F**) Schematic diagram of the reaction mechanism for the oxidation of glycerol to formic acid (with the gray box representing O-H bond activation, the blue box representing C–H bond activation, and the orange box representing the hydroxyl radical reaction and C–C bond cleavage). Reproduced with permission: Copyright 2023, Springer Nature [51].

For instance, the PtCu catalysts, particularly in the form of single-atom alloys (SAA), play a pivotal role in the selective hydrogenolysis of glycerol to 1,2-propanediol (1,2-PDO) [115]. These catalysts exhibit a bimetallic synergy, where single Pt atoms dispersed on Cu surfaces create unique active sites. Specifically, the Pt atoms facilitate the activation of the central C–H bonds in glycerol, promoting its dehydrogenation, while the Cu atoms are adept at activating and cleaving the terminal C–O bonds. This interaction at the Pt–Cu interface significantly lowers the activation energy barrier, thereby enhancing both the catalytic activity and selectivity towards 1,2-PDO under mild conditions (Figure 5A,B). The effectiveness of PtCu catalysts stems from their ability to alter the electronic environment at the catalytic sites, thus stabilizing reaction intermediates and accelerating the reaction kinetics. Building on the versatility of platinum in catalysis, Pt_1_ catalysts, which consist of isolated Pt atoms, are highly effective in the initial activation of glycerol through C–H bond cleavage [107]. These single atoms offer a unique electronic structure that enables precise and efficient dehydrogenation of glycerol to intermediate products such as glyceraldehyde. Complementing this, Pt_n_ catalysts, composed of small Pt clusters, enhance the subsequent steps by facilitating the activation of O–H bonds and the oxidation of intermediates into more oxidized forms, such as glyceric acid. The distinct catalytic roles of Pt_1_ and Pt_n_ arise from their ability to stabilize different transition states and reaction intermediates, leading to a highly efficient oxidation process (Figure 5C,D). Therefore, the combination of Pt_1_ and Pt_n_ in a catalytic system allows for a cascade oxidation mechanism, where Pt_1_ drives the initial dehydrogenation and Pt_n_ completes the oxidation sequence. In contrast to platinum-based systems, manganese dioxide (MnO_2_) catalysts, especially those that are defect-rich, offer a different approach to glycerol activation, focusing on oxidation. Defective MnO_2_ catalysts, characterized by the presence of unsaturated Mn^δ+^ sites and abundant oxygen vacancies, serve as powerful catalysts for the oxidation of glycerol to formic acid [51]. The defects in MnO_2_ create Frustrated Lewis Pairs (FLPs), where the Mn^δ+^ sites act as Lewis acids, facilitating the activation of molecular oxygen (O_2_), while the adjacent oxygen vacancies function as Lewis bases, enhancing the activation of C–H and C–C bonds in glycerol (Figure 5E,F). These FLPs synergistically promote both the adsorption and activation of O_2_ and glycerol, leading to more efficient oxidative cleavage of C–C and C–H bonds. The defect-rich structure in MnO_2_ significantly improves the catalytic turnover frequency and selectivity towards formic acid, rivaling that of noble metal catalysts. Thus, the high catalytic performance of MnO_2_ is attributed to its ability to stabilize reactive oxygen species and reaction intermediates, which are crucial for the selective oxidation of glycerol.

In summary, metal-based catalysts such as PtCu, Pt_1_/Pt_n_, and MnO_2_ are integral to driving the efficient activation and transformation of glycerol through various mechanistic pathways. The PtCu catalysts harness the synergistic effects of Pt and Cu to lower activation energies and enhance selectivity, while Pt_1_ and Pt_n_ catalysts leverage their atomic and cluster configurations to drive sequential oxidation steps. Meanwhile, MnO_2_ catalysts, with their defect-rich structures, provide a robust platform for the oxidative cleavage of glycerol, offering a high degree of control over the reaction kinetics and product distribution. Collectively, these metal-based catalysts enable the precise tuning of glycerol conversion processes, leading to the efficient production of valuable chemicals under mild reaction conditions.

### 3.3. Formation and Stabilization of Intermediates

The formation and stabilization of intermediates are vital in the metal-catalyzed oxidation of glycerol, significantly influencing the efficiency and selectivity of the overall process. Glycerol, being a versatile and renewable feedstock, undergoes various transformations facilitated by metal catalysts to produce numerous high-value chemicals. These transformations hinge on the ability of catalysts to stabilize key intermediates such as glyceraldehyde and dihydroxyacetone, which are crucial for subsequent reactions, leading to valuable products like lactic acid. Metal catalysts, including iridium and bismuth-based compounds, play a crucial role in promoting the initial dehydrogenation of glycerol to DHA and GAL, preventing overoxidation and minimizing carbon–carbon bond cleavage. This selective stabilization ensures that intermediates like DHA and GAL proceed through desired pathways, enhancing the overall efficiency and selectivity of the glycerol oxidation process. Understanding the mechanisms by which these intermediates are formed and stabilized provides valuable insights for optimizing catalytic systems, leading to higher yields and selectivity in glycerol oxidation. This knowledge is fundamental for advancing sustainable chemical processes, leveraging renewable feedstocks like glycerol to produce high-value chemicals.

**Figure 6:**
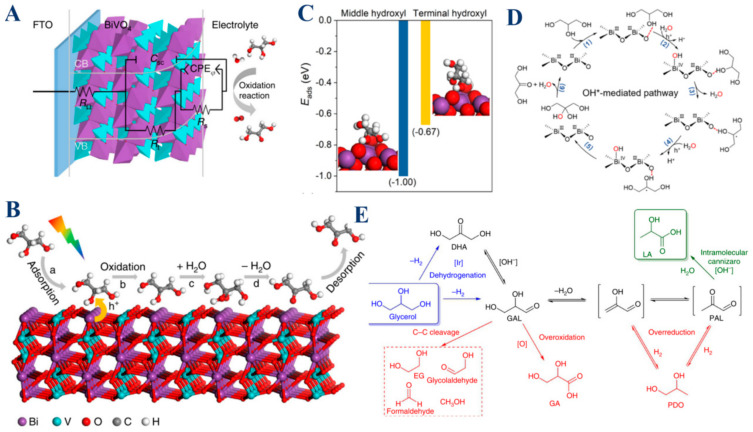
(**A**) Equivalent circuit employed to conceptualize the BiVO_4_ photoanode during PEC reactions. (**B**) Schematic illustration depicting PEC glycerol oxidation to DHA. Reproduced with permission: Copyright 2019, Springer Nature [15]. (**C**) Adsorption energies of the glycerol with its middle hydroxyl or terminal hydroxyl adsorbed on Bi_2_O_3_ (201). The optimized adsorption geometries are also displayed. The color of each element is violet for Bi, red for O, white for H, and gray for C, respectively. The dashed blue line represents the hydrogen bond between glycerol and Bi_2_O_3_ (201). (**D**) Proposed OH*-mediated pathway. Reproduced with permission: Copyright 2022, American Chemical Society [19]. (**E**) Proposed mechanism for converting glycerol to lactic acid (depicted in green) and other common byproducts (shown in red). Reproduced with permission: Copyright 2014, Springer Nature [44].

Among these catalysts, BiVO_4_ stands out as an effective photoanode in the selective photoelectrochemical oxidation of glycerol. When illuminated, BiVO_4_ generates photogenerated holes that are instrumental in oxidizing adsorbed glycerol into radicals, key intermediates in the production of DHA [15]. The structure of BiVO_4_, particularly its capacity to efficiently adsorb glycerol at low pH levels, plays a crucial role in enhancing the formation and stabilization of these radicals. Furthermore, BiVO_4_ favors the formation of a stable tertiary radical on the middle carbon atom of glycerol, a step that is crucial for the selective production of DHA (Figure 6A,B). This strong interaction between glycerol and the BiVO_4_ surface, especially in acidic environments, not only stabilizes the intermediates but also ensures that the oxidation process is efficiently directed toward the desired product without overoxidation or the formation of secondary byproducts. Thus, BiVO_4_ exemplifies how a well-designed catalyst can effectively balance intermediate formation and stabilization to achieve high selectivity, making it a key player in glycerol oxidation. Similarly, Bi_2_O_3_/TiO_2_ plays a crucial role in both the formation and stabilization of reaction intermediates, particularly for the selective conversion of glycerol to dihydroxyacetone (DHA) [19]. The Bi_2_O_3_ nanoparticles, when supported on TiO_2_ nanorods, enhance the overall photoelectrocatalytic (PEC) performance by forming a p-n junction, which promotes charge transfer and increases photocurrent density. This setup also enhances optical absorption, further boosting the PEC activity. One of the key functions of Bi_2_O_3_ is its preferential interaction with the middle hydroxyl group of glycerol. This interaction facilitates the selective oxidation of glycerol to DHA. The study revealed that the glycerol oxidation proceeds through two main pathways: one mediated by electrophilic OH* radicals (the major pathway) and another by lattice oxygen (the minor pathway). The Bi_2_O_3_ component is critical for the generation and stabilization of OH* radicals on its surface, which selectively oxidize glycerol to DHA while minimizing overoxidation and the formation of unwanted byproducts (Figure 6D). The stabilization of these intermediates is essential to maintaining high selectivity towards DHA, even at relatively high conversion rates. Moreover, Bi_2_O_3_’s ability to preferentially adsorb the middle hydroxyl group of glycerol while allowing easier desorption of the product DHA prevents further oxidation, which is pivotal in achieving high DHA selectivity. The combined effects of enhanced charge transfer, selective adsorption, and controlled oxidation make Bi_2_O_3_/TiO_2_ an effective catalyst for the selective photoelectrocatalytic oxidation of glycerol. In contrast to the previous examples, iridium catalysts, especially Cp*Ir complexes, offer a different approach to selective glycerol oxidation [44]. These catalysts are exceptional in catalyzing the dehydrogenation of glycerol, leading to the formation of intermediates such as DHA or glyceraldehyde (GAL). These intermediates are then further processed under mild conditions to produce lactic acid, a valuable chemical product. The iridium catalyst’s role extends beyond merely forming these intermediates, and it also plays a crucial role in stabilizing them, ensuring that they follow the intended reaction pathway toward lactic acid formation. This stabilization is critical in preventing the intermediates from decomposing or converting into other, less desirable compounds, thereby maintaining the high selectivity and efficiency of the oxidation process (Figure 6E). Moreover, the ability of iridium catalysts to operate under mild conditions without the need for external hydrogen or oxygen further underscores their effectiveness in stabilizing these intermediates and driving the selective oxidation of glycerol. Thus, iridium catalysts demonstrate how careful control over reaction conditions and catalyst design can lead to highly selective and efficient oxidation processes.

In summary, metal-based catalysts such as Bi_2_O_3_/TiO_2_, iridium complexes, and BiVO_4_ are integral to the selective oxidation of glycerol. Their ability to form and stabilize reaction intermediates ensures high selectivity and efficiency in producing valuable chemical products like DHA and lactic acid. By enhancing charge transfer, controlling reaction pathways, and stabilizing key intermediates, these catalysts collectively demonstrate their vital role in advancing the field of glycerol oxidation. Understanding the nuanced roles of these catalysts not only highlights their individual strengths but also provides insights into designing more effective catalytic systems for future applications. Through their shared and unique mechanisms, these metal-based catalysts are driving innovations in selective oxidation processes, paving the way for more sustainable and efficient chemical transformations.

### 3.4. Selective C–C Bond Cleavage

The selective cleavage of carbon–carbon (C–C) bonds in glycerol oxidation is a critical mechanistic pathway for converting glycerol into valuable chemical products. This process is highly significant due to its potential to generate smaller, industrially important molecules through the targeted breaking of C–C bonds in the glycerol molecule. The primary products formed through this pathway include glycolaldehyde and other C2 species, which serve as crucial intermediates in the synthesis of various high-value chemicals. Recent studies have demonstrated that metal catalysts play a significant role in facilitating selective C–C bond cleavage. By understanding the mechanistic pathways and the roles of various metal catalysts, researchers can develop more efficient and selective processes for converting glycerol into high-value chemicals, thereby enhancing the economic and environmental benefits of glycerol utilization.

**Figure 7:**
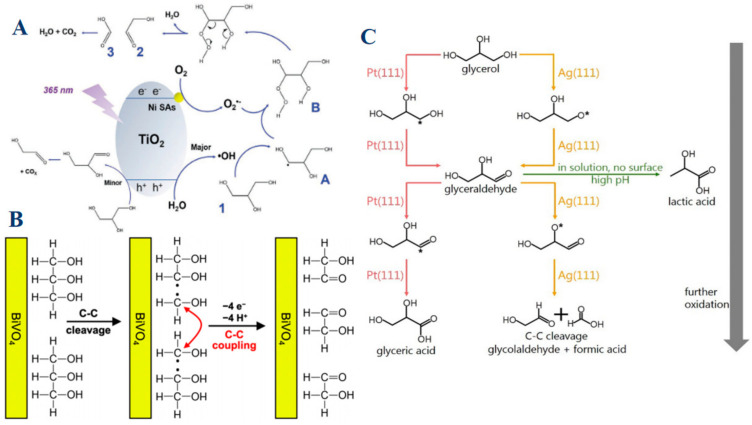
(**A**) Proposed reaction mechanism for glycerol oxidation on Ni single-atom decorated TiO_2_. Reproduced with permission: Copyright 2023, Wiley-VCH [54]. (**B**) Schematic example showing how C−C cleavage can be coupled with extraction of an electron/proton pair. The resulting C1 radical can be further oxidized to form a C1 product like FAD or FA, or it can undergo C−C coupling to form a C2 species. Reproduced with permission: Copyright 2023, American Chemical Society [18]. (**C**) Proposed pathways for glycerol electro-oxidation on Pt (111) and Ag (111) surfaces. Gibbs free energy difference for glycerol adsorption (C_3_H_8_O_3_(g) → C_3_H_7_O_3_* + 1/2H_2_(g)) through C* adsorption (ΔGC site) compared to O* adsorption (ΔGO site) on (111) metal surfaces. Reproduced with permission: Copyright 2024, American Chemical Society [83].

Recent studies have demonstrated that metal catalysts play a significant role in facilitating selective C–C bond cleavage. For instance, nickel-based catalysts, especially when employed as single atoms dispersed on titanium dioxide (TiO_2_), exhibit remarkable catalytic properties in the selective oxidation of glycerol, particularly in promoting C–C bond cleavage [54]. Ni atoms serve as active sites for oxygen adsorption and activation, a crucial step that leads to the formation of superoxide radicals (O_2_•^−^). These radicals are highly reactive and promote the targeted cleavage of C–C bonds in glycerol, leading to the formation of products like glycolaldehyde (Figure 7A). Additionally, Ni atoms act as electron sinks, facilitating effective charge separation and reducing the recombination of photogenerated charge carriers. This enhances the overall efficiency of the oxidation process. The unique electronic environment provided by single Ni atoms on TiO_2_ also enables the generation of specific reactive oxygen species, which are instrumental in driving the selective oxidation of glycerol to desired products. Building upon this understanding, the role of BiVO_4_ introduces an additional layer of complexity, demonstrating how photoelectrochemical conditions and unique band structures can drive not only selective C–C bond cleavage but also facilitate C–C coupling reactions that further optimize product yield [18]. BiVO_4_ is another metal oxide catalyst that demonstrates a profound impact on glycerol oxidation, especially under photoelectrochemical conditions. BiVO_4_ excels not only in promoting C–C bond cleavage but also in enabling C–C coupling reactions, which are relatively rare in other catalytic systems (Figure 7B). This dual capability significantly boosts the yield of glycolaldehyde, a key oxidation product. The efficiency of BiVO_4_ in this process is largely due to its ability to effectively utilize photogenerated holes, which are crucial for driving the oxidation reactions. Furthermore, BiVO_4_ exhibits a high degree of selectivity for glycolaldehyde production under both acidic and alkaline conditions. Its poor kinetics for the oxygen evolution reaction (OER) are advantageous in this context, as they minimize competition from water splitting, ensuring that the majority of photogenerated holes are directed towards glycerol oxidation. While Ni and BiVO_4_ offer insights into the manipulation of catalytic environments to favor specific oxidation pathways, the comparison of Pt (111) and Ag (111) surfaces provides a contrasting perspective [83]. Platinum (Pt) and silver (Ag) catalysts, specifically their Pt (111) and Ag (111) surfaces, offer a striking contrast in their catalytic behaviors due to differences in their adsorption preferences and electronic structures. Pt (111) surfaces favor the adsorption of glycerol and its intermediates via carbon atoms (C*), leading to the formation of larger, more complex C3 products such as glyceraldehyde and glyceric acid (Figure 7C). The pathway on Pt (111) involves sequential oxidation steps that generally preserve the C–C bonds, with C–C bond cleavage occurring only under strong oxidizing conditions or high potentials. This makes Pt (111) highly selective for producing C3 products, with glyceraldehyde serving as a key intermediate. In contrast, Ag (111) surfaces exhibit a preference for oxygen atom (O*) adsorption, which significantly alters the oxidation pathway. On Ag (111), glycerol oxidation tends to favor the cleavage of C–C bonds, leading to the formation of smaller products such as glycolaldehyde and formic acid. This behavior is driven by the distinct electronic structure of Ag, which facilitates the breaking of C–C bonds during the oxidation process. The preference of Ag (111) for oxygen adsorption not only dictates the reaction pathway but also results in a higher selectivity for C2 and C1 products, making Ag (111) particularly effective for processes where smaller oxidation products are desired.

Overall, the catalytic performance and selectivity of metal-based catalysts in glycerol oxidation are intricately linked to their electronic properties, surface adsorption behaviors, and the types of reactive intermediates they stabilize. By understanding these relationships, it is possible to tailor the catalytic process to favor specific reaction pathways, whether the aim is to preserve C–C bonds and produce larger molecules or to promote C–C bond cleavage for the generation of smaller, more oxidized products. The choice of metal catalyst, therefore, plays a crucial role in determining the efficiency and selectivity of glycerol oxidation, making it a key factor in the design of catalytic systems for industrial applications.

## 4. Conclusions and Outlook

Advancing the field of glycerol oxidation necessitates focused research efforts to overcome current limitations and explore new opportunities. Key areas for future research include the development of innovative catalysts, detailed mechanistic studies, and the scalability of industrial applications.

Developing innovative catalysts with enhanced efficiency and selectivity is crucial for improving glycerol oxidation processes. Future research should prioritize creating single-atom catalysts and multi-metallic systems, which have shown potential for superior catalytic performance due to their unique electronic and geometric properties. Single-atom catalysts can provide precise control over reaction pathways, resulting in higher selectivity for desired products. Additionally, exploring novel support materials, such as advanced carbon materials and metal–organic frameworks (MOFs), can significantly enhance the dispersion of active sites, increase stability, and improve overall catalytic performance [67,121]. These supports can also influence the electronic properties of the active metals, further boosting their activity and selectivity.

A deeper understanding of the catalytic mechanisms and intermediate species involved in glycerol oxidation is essential for designing more effective catalysts. Future research should employ advanced spectroscopic and computational techniques to gain these insights [110,122,123]. In situ techniques, such as X-ray absorption spectroscopy, nuclear magnetic resonance spectroscopy, and infrared spectroscopy, can provide real-time information on the structure and electronic state of catalysts during the reaction. Computational studies using density functional theory calculations and molecular dynamics simulations can offer detailed insights into the electronic and geometric effects that influence catalyst performance. By investigating the role of these effects on catalyst behavior, researchers can fine-tune catalyst design to optimize reaction outcomes.

Translating laboratory-scale successes into industrial applications poses significant challenges that need to be addressed. Future research should focus on scaling up the most promising catalytic systems for industrial applications, ensuring that these processes are economically viable and environmentally sustainable. This involves optimizing reaction conditions, such as temperature, pressure, and reactant concentrations, to achieve high turnover frequencies and catalyst lifetimes on an industrial scale. Furthermore, integrating glycerol oxidation processes into existing biorefineries and chemical production facilities can enhance overall sustainability. This integration can create synergies with other processes, improving resource utilization and reducing waste. Conducting life cycle assessments and techno-economic analyses can also evaluate the economic and environmental benefits of large-scale glycerol oxidation processes, identifying potential bottlenecks and areas for improvement.

Adopting sustainable and green chemistry principles is essential for the future of glycerol oxidation research. Key strategies include using renewable feedstocks and minimizing waste, fundamental principles of green chemistry. Glycerol, as a byproduct of biodiesel production, represents a renewable and abundant feedstock for chemical synthesis. Developing catalytic systems that operate under mild conditions, such as low temperatures and pressures, and using water or green solvents instead of toxic or hazardous chemicals, can reduce the environmental footprint of glycerol oxidation processes. Additionally, designing catalysts that can be easily recovered and reused can improve the sustainability of the oxidation process. Research should focus on developing stable and durable catalysts that maintain high activity and selectivity over multiple cycles.

Beyond traditional metal-catalyzed oxidation, exploring new catalytic pathways can open novel possibilities for glycerol valorization. Future research should investigate photocatalysis, electrocatalysis, and biocatalysis in glycerol oxidation. Photocatalytic systems that utilize visible light for activating catalysts can reduce energy consumption, while electrocatalytic approaches can provide precise control over reaction pathways through applied potentials. Biocatalysis, involving enzymes or whole cells, can offer highly selective and mild conditions for glycerol conversion. Combining these approaches with traditional metal catalysis can lead to the development of hybrid catalytic systems with enhanced performance.

In conclusion, future research in glycerol oxidation should focus on developing innovative catalysts, conducting detailed mechanistic studies, scaling up processes for industrial applications, adopting sustainable practices, and exploring new catalytic pathways. These efforts will contribute to the advancement of efficient, selective, and environmentally friendly processes for the valorization of glycerol, supporting the broader goals of sustainable chemistry and industrial innovation.

## Figures and Tables

**Table 1:** Summary of analytical techniques, glycerol oxidation, and byproduct analysis for different catalysts.

Catalyst	Analytical Techniques	Products	Highly Selective Product	References
WO_3_	HPLC	Glyceric acid (73%), Dihydroxyacetone (23%), Formic acid (4%)	Glyceric acid Selectivity: 73 %	[110]
Mn-3	^1^H NMR	1,2-propanediol (2%), Lactic acid (98%)	Lactic acid Selectivity: 98%	[82]
CoO/NF	HPLC	Glyceraldehyde (0.6%), Glyceric acid (0.36%), Glycolic acid (2.8%), Oxalic acid (2.94%), Formic acid (93.3%)	Formic acid Selectivity: 93.3%	[88]
Ni_1_Co_1_O*_x_*	HPLC	Glyceric acid (66.5%), Glycolic acid (19.5%), Oxalic acid (6.4%)	Glyceric acid Selectivity: 66.5%	[91]
Pt–Bi/SBA-15	HPLC GC	Dihydroxyacetone (65.1%), Glyceric acid (4.7%), Hydroxypyruvic acid (11.1%), Mesoxalic acid (6.8%)	Dihydroxyacetone Selectivity: 65.1%	[111]
Bi-rich BiVO_4−x_	HPLC	Dihydroxyacetone (80.3%), Glyceric acid (7.5%), Glyceraldehyde, Glycolic acid (2.05%), Formic acid (10.1%)	Dihydroxyacetone Selectivity: 80.3%	[16]
0.9%Pt_1_ + Pt_n_/Cu − CuZrO_x_	GC HPLC	Acetic acid (0.7%), Glyceraldehyde (4.6%), Dihydroxyacetone (10.5%), Glyceric acid (80%), Tartronic (1.4%), Oxalic acid (1.2%), Glycolic acid (1.1%)	Glyceric acid Selectivity: 80%	[107]
MnO_2_-D	HPLC	Glyceric acid (1.4%), GLYOA (3.2%), XA (2.8%), Formic acid (83.2%)	Formic acid Selectivity: 83.2%	[51]
BiVO_4_	LC-MS	Formic acid (16.4%), Glyceric acid (10.0%), Dihydroxyacetone (63.6%), Glycolic acid (0.2%), CO_2_ (0.9), CO (0.2%)	Dihydroxyacetone Selectivity: 63.6%	[15]
Bi_2_O_3_/TiO_2_	HPLC	Dihydroxyacetone (65%), Glycolic acid (4%), Glyceraldehyde (12.7%), Formic acid (7.8%)	Dihydroxyacetone Selectivity: 65%	[19]
Catalyst 16	^1^H NMR	1,2-propanediol (0.6%), Ethylene glycol (2.4%), Formic acid (97%), Formic acid (0.1%)	Lactic acid Selectivity: 97%	[44]
0.5Ni/TiO_2_−MS	HPLC	Glycolaldehyde, Glyceraldehyde, Dihydroxyacetone, Formic acid	Glycolaldehyde Selectivity: 60.1%	[56]
BiVO_4_	HPLC	Glycolaldehyde (60%), Formic acid (27%), Glyceraldehyde (5%), Glyceric acid (1%), Dihydroxyacetone (6%), Glycolic acid (1%)	Glycolaldehyde, Selectivity: 60%	[18]

## Data Availability

No new data were created or analyzed in this study.
